# Urinary incontinence in pregnant women and its impact on health-related quality of life

**DOI:** 10.1186/s12955-022-01920-2

**Published:** 2022-01-21

**Authors:** Xiaojuan Wang, Ying Jin, Ping Xu, Suwen Feng

**Affiliations:** 1 Zhejiang University School of Medicine, No.866 Yu Hang Tang Road, Hangzhou, 310058 Zhejiang Province China; 2 Women’s Hospital, School of Medicine Zhejiang University, No.1 Xue Shi Road, Hangzhou, 310006 Zhejiang Province China

**Keywords:** Health-related quality of life, Help-seeking behavior, Pregnancy, Risk factor, Urinary incontinence

## Abstract

**Background:**

Urinary incontinence is a common and distressing condition affecting women worldwide. However, urinary incontinence during pregnancy was less studied. The study aims to investigate the prevalence and risk factors of urinary incontinence during pregnancy, its impact on health-related quality of life as well as associated help-seeking behavior.

**Methods:**

Eligible women were enrolled in the obstetric wards of a tertiary maternity hospital. Urinary incontinence, generic and specific health-related quality of life were assessed using the International Consultation on Incontinence Modular Questionnaire-Urinary Incontinence Short Form (ICIQ-UI SF), the 12-Item Short Form Health Survey version 2 (SF-12v2), Urogenital Distress Inventory short form (UDI-6) and Incontinence Impact Questionnaire short form (IIQ-7), respectively. Multiple logistic regression and multiple linear regression analysis were used to examine risk factors of urinary incontinence during pregnancy and the impact of incontinence on health-related quality of life of pregnant women, respectively.

**Results:**

A total of 1243 women were enrolled in the study. The prevalence of urinary incontinence during pregnancy was 52.0%. Most women suffered from mild or moderate incontinence. Five risk factors were identified by multiple logistic regression. Urinary incontinence before pregnancy was the strongest predictor for incontinence during pregnancy (OR = 4.178, 95% CI = 2.690–6.490), followed by history of vaginal birth, coffee consumption, childhood enuresis and history of urinary tract infection. Urinary incontinence had significant impact on health-related quality of life during pregnancy. Only 14.8% of pregnant women sought professional help for urinary symptoms.

**Conclusions:**

Urinary incontinence was highly prevalent in pregnant women, with a broad detrimental effect on health-related quality of life. Five factors were confirmed to be associated with increasing the risk of developing urinary incontinence during pregnancy. The help-seeking behavior during pregnancy was discouraging. Targeted interventions are warranted to facilitate the prevention of urinary incontinence and improvement of health-related quality of life in pregnant women.

## Background

Urinary incontinence is a common condition in women worldwide, which can be distressing both physically and mentally and burdensome to the individual and society [1]. Many women may first experience urinary incontinence during pregnancy. Physiological changes during pregnancy such as increased abdominal pressures and progesterone levels and injury to the pelvic floor may make women more susceptible to urinary incontinence [2]. As a result, more than half of women suffered from urinary incontinence during pregnancy and peaked in the third trimester [3–6]. Urinary incontinence during pregnancy was a strong predictor of urinary incontinence postpartum and later in life even for those who recovered immediately after birth [1, 7]. In comparison with urinary incontinence in middle-aged and older women, risk factors of urinary incontinence during pregnancy were less studied and results were inconsistent [3, 5, 6, 8, 9]. Identifying risk factors of urinary incontinence during pregnancy will inform decision making for health care providers and pregnant women. Then, effective preventive strategies without adverse effects such as pelvic floor muscle training can be targeted during pregnancy to prevent urinary incontinence in late pregnancy and postnatal period [10].

Urinary incontinence is not a life-threatening condition but could have a quite broad detrimental impact on health-related quality of life including physical, psychological and social well-being compared with other diseases [11]. On the one hand, women with urinary incontinence may rush to the bathroom increasing the risk of falls and fractures [12]. In many cases, mobility (the ability to participate in exercise, travel and so on) could be affected by incontinence because many women tended not to go out as a result of fear of urinary leakage [11]. Besides, urinary incontinence was linked to sleep disturbances and urinary tract infections [13]. On the other hand, there is evidence that psychological and social function were affected the most by incontinence. The distress such as shame and embarrassment caused by urinary incontinence may compel women to choose a solitary life and even develop depression [14]. More importantly, different aspects of quality of life were mutually influenced by each other. However, studies regarding the impact of incontinence on quality of life in pregnant women were limited and primarily focused on specific quality of life [4, 15–17]. In addition, health-related quality of life is culturally specific, the results in one country cannot be generalized to another country.

Despite the high prevalence and negative impact on individual’s daily life across many aspects, only a few women sought medical help for urinary symptoms. Zhu et al. reported that only 25% of adult women sought medical help for urinary symptoms in China [18]. Pregnancy is a very special period for women. Consulting the doctor about urinary symptoms could help pregnant women get a deeper understanding of incontinence and alleviate symptoms by taking effective preventive strategies timely. So far, only one study regarding help-seeking behavior of pregnant women was conducted and indicated that only a few women sought help for incontinence during pregnancy [19]. Besides, there is a wide disparity regarding the reasons underlying help-seeking behaviors for incontinence in different countries as a result of different sociocultural background [20]. To date, the results of epidemiological studies of incontinence during pregnancy were inconsistent and studies exploring the effect of incontinence on health-related quality of life as well as help-seeking behavior were scarce. The study aims to investigate the prevalence and risk factors of urinary incontinence during pregnancy, its impact on generic and specific quality of life as well as incontinence related help-seeking behavior, which may provide valuable insight for the prevention of incontinence during pregnancy and improvement of quality of life. We hypothesized that (1) demographic variables, obesity, caffeine intake, incontinence related history, gestational diabetes mellitus and mode of delivery could contribute to incontinence during pregnancy; (2) urinary incontinence could have a broad detrimental effect on health-related quality of life but with a low help-seeking rate.

## Methods

This was a cross-sectional study, which was approved by the Hospital Institutional Review Board (No. 20180080). The study protocol was in accordance with the Declaration of Helsinki. Written fully informed consent was obtained from participants and data were guaranteed to be confidential. The report was in consistent with the STROBE statement.

### Participants

Women aged 18 years or older with a singleton and term pregnancy were consecutively recruited in the obstetric wards of a university-affiliated tertiary maternity hospital in Hangzhou, a provincial capital city in eastern China, from January to June 2020. As one of the largest maternity hospitals in China, it has 1120 beds and offers a range of healthcare services for women, delivering over 20,000 babies a year. Women were excluded from the study if they had: (1) active urinary tract infection; (2) stillbirth; (3) fetus with congenital malformation; (4) Severe comorbidity including severe cardiopulmonary disease, kidney disease, liver disease, cerebrovascular disease or other diseases, which may make women too weak to complete the survey. Both the inclusion and exclusion criteria were assessed based on medical records using ICD-10 diagnosis codes.

### Measurements

In the study, candidate risk factors (demographics, coffee consumption, past medical history, etc.), help-seeking behavior and bother were assessed with a self-designed questionnaire. Urinary incontinence and health-related quality of life were assessed with standardized questionnaires. Data were collected by trained researchers through a pencil-and-paper survey.

Urinary incontinence was measured with the International Consultation on Incontinence Modular Questionnaire-Urinary Incontinence Short Form (ICIQ-UI SF), which was used for evaluating the prevalence, severity and type of urinary incontinence [21]. Incontinence severity can be divided into four categories according to the total score of the first three items of ICIQ-UI SF: slight (0–5), moderate (6–12), severe (13–18) and very severe (19–21) [22]. The Chinese version of ICIQ-UI SF has been validated with adequate reliability and validity [23].

Generic health-related quality of life was assessed with the 12-item Short Form Health Survey version 2 (SF-12v2), which consists of eight domain scales: physical functioning, role limitation due to physical problems, bodily pain, general health, vitality, social functioning, role limitation due to emotional problems and mental health. Domain scales are aggregated into two component summary measures: physical component summary and mental component summary, indicating physical health and mental health, respectively. All domain scales and component summary scores range from 0 to 100, with higher score suggesting better health-related quality of life. The SF-12v2 has been validated in Chinese population with good psychometric properties [24].

Specific health-related quality of life was assessed with the Urogenital Distress Inventory short form (UDI-6) and Incontinence Impact Questionnaire short form (IIQ-7), consisting of six and seven items, respectively [25]. The total score ranges from 0 to 100. The higher the score, the worse the quality of life. The scales have been validated in Chinese population with adequate psychometric properties [26].

The help-seeking behavior was a dichotomous variable, which was measured with the question: “Have you ever sought help from health care providers such as doctors and nurses due to urinary leakage during pregnancy?” The bother of incontinence was assessed with the question: “How much does urinary leakage bother your life?” Responses were divided into four categories (not at all, mildly, moderately and severely).

### Statistical analysis

The sample size was calculated according to the formula n = 1.96^2^ *p*(1 − *p*)(*DEFF*)/*d*^2^ [27]. In this study, *p* was 50% since the prevalence of urinary incontinence was assumed to be 50% based on previous studies [3, 4, 6]. The *d* value was ± 5% for estimated prevalence in the range of 20–80%. The *DEFF* was estimated to be 2 due to non-random sampling. Hence, the sample size required to assure statistical power was 769.

Descriptive analysis was performed to describe the characteristics of participants, the types, severity and bother of urinary incontinence, health-related quality of life and help-seeking behavior. An independent *t*-test and chi-square test were used to detect differences in candidate predictors between groups for continuous variables conforming to normal distribution and categorical variables, respectively. Multiple logistic regression was applied to examine risk factors of urinary incontinence during pregnancy. Multiple linear regression analysis was used to examine the impact of incontinence on health-related quality of life of pregnant women. Statistical analysis was performed using SPSS software, version 22.0 (IBM Corp., Armonk, NY). A *p*-value less than 0.05 was considered statistically significant.

## Results

A total of 1243 women were included for analysis, amongst whom 80.4% lived in city and 86.6% were 35 years old or younger. The characteristics of all participants and comparison between women with and without incontinence were presented in Table 1. In the study, 646 (52.0%) women suffered from urinary incontinence during pregnancy, with stress incontinence of 75.4%, urgency incontinence of 5.3% and mixed incontinence of 7.9%. Most women suffered from mild or moderate incontinence and were not greatly bothered by urinary leakage. Incontinence related variables were shown in Table 2.

**Table 1:** Characteristics of participants (n = 1243)

Variables	All sample	women with UI	women without UI	*P*
Age (years)				
≤ 35	1077 (86.6%)	543 (50.4%)	534 (49.6%)	0.005
> 35	166 (13.4%)	103 (62.0%)	63 (38.0%)	
Residence				
City	1000 (80.4%)	530 (53.0%)	470 (47.0%)	0.141
Country	243 (19.6%)	116 (47.7%)	127 (52.3%)	
Education				
Junior college or below	444 (35.7%)	233 (52.5%)	211 (47.5%)	0.773
Bachelor degree	625 (50.2%)	327 (52.2%)	298 (47.8%)	
Master degree or above	174 (14.1%)	86 (49.4%)	88 (50.6%)	
Coffee consumption				
< Once a week	1164 (93.6%)	595 (51.1%)	569 (48.9%)	0.021
≥ Once a week	79 (6.4%)	51 (64.6%)	28 (35.4%)	
Tea consumption				
< Once a week	1157 (93.1%)	595 (51.4%)	562 (48.6%)	0.158
≥ Once a week	86 (6.9%)	51 (59.3%)	35 (40.7%)	
Childhood enuresis				
No	1130 (90.9%)	573 (50.7%)	557 (49.3%)	0.005
Yes	113 (9.1%)	73 (64.6%)	40 (35.4%)	
Family history of UI				
No	1187 (95.5%)	612 (51.6%)	575 (48.4%)	0.180
Yes	56 (4.5%)	34 (60.7%)	22 (39.3%)	
Gestational diabetes mellitus				
No	959 (77.2%)	498 (51.9%)	461 (48.1%)	0.957
Yes	284 (22.8%)	148 (52.1%)	136 (47.9%)	
History of urinary tract infection				
No	1052 (84.6%)	525 (49.9%)	527 (50.1%)	0.001
Yes	191 (15.4%)	121 (63.4%)	70 (36.6%)	
Constipation				
No	1032 (83.0%)	533 (51.6%)	499 (48.4%)	0.613
Yes	211 (17.0%)	113 (53.6%)	98 (46.4%)	
Pre-pregnancy body mass index	21.1 ± 2.8	21.2 ± 2.8	21.0 ± 2.7	0.157
History of childbirth				
No	788 (63.4%)	380 (48.2%)	408 (51.8%)	< 0.001
Cesarean section	230 (18.5%)	112 (48.7%)	118 (51.3%)	
Vaginal birth	225 (18.1%)	154 (68.4%)	71 (31.6%)	
UI before pregnancy				
No	1088 (87.5%)	519 (47.7%)	569 (52.3%)	< 0.001
Yes	155 (12.5%)	127 (81.9%)	28 (18.1%)	

*UI* urinary incontinence

**Table 2:** Types, severity and bother of urinary incontinence during pregnancy (n = 646)

Variables	n (%)
Types of urinary incontinence	
Stress urinary incontinence	487 (75.4%)
Urgency urinary incontinence	34 (5.3%)
Mixed urinary incontinence	51 (7.9%)
Other types	74 (11.4%)
Severity of urinary incontinence	
Slight (0–5)	358 (55.4%)
Moderate (6–12)	268 (41.5%)
Severe (13–18)	19 (2.9%)
Very severe (19–21)	1 (0.2%)
Bother of urinary incontinence	
No	170 (26.3%)
Mild	428 (66.3%)
Moderate	35 (5.4%)
Severe	13 (2.0%)

As shown in Table 1, thirteen variables were included as candidate risk factors based on literature review and clinical reasoning, amongst which ten variables with *p* value ≤ 0.2 in univariate analysis were included for multivariate analysis. Finally, five risk factors were identified by multiple logistic regression (Table 3). Urinary incontinence before pregnancy was the strongest predictor for incontinence during pregnancy (OR = 4.178, 95% CI = 2.690–6.490), followed by history of vaginal birth, coffee consumption, childhood enuresis and history of urinary tract infection.

**Table 3:** Logistic regression analysis of risk factors for urinary incontinence during pregnancy

Variables	OR (95%CI)	*p*
Coffee consumption	1.763 (1.078–2.884)	0.024
Childhood enuresis	1.616 (1.059–2.465)	0.026
History of urinary tract infection	1.502 (1.077–2.096)	0.017
UI before pregnancy	4.178 (2.690–6.490)	< 0.001
History of childbirth		
No	Reference	
History of cesarean section	0.858 (0.630–1.170)	0.335
History of vaginal birth	1.894 (1.364–2.629)	< 0.001

*UI* urinary incontinence

As shown in Table 4, women with urinary incontinence showed significant lower score in three domain scales of mental health (social functioning, role-emotional, mental health) and mental component summary (49.6 ± 8.1 vs. 51.5 ± 7.7, *p* < 0.001), indicating more interference with normal social activities, more limitations in work and other activities due to emotional problems and more negative feelings of nervousness and depression. In addition, a significant lower score was observed in two domain scales of physical health (general health, role-physical) in women suffering from incontinence, which denoted more negative evaluation of general health and more problems in work and daily activities as a result of physical problems. The scores of IIQ-7 and UDI-6 were shown in Table 4. The median of IIQ-7 was 4.8 (0–100) and the average score of UDI-6 was 23.0 (0–100). The more severe the incontinence symptoms, the worse the quality of life perceived by pregnant women.

**Table 4:** The impact of urinary incontinence on health-related quality of life of pregnant women

Variables	Women with UI	Women without UI	*p*
SF-12v2			
Physical component summary	44.4 ± 7.4	45.0 ± 7.7	0.215^a^
Mental component summary	49.6 ± 8.1	51.5 ± 7.7	< 0.001^a^
Physical functioning	44.1 ± 9.9	44.1 ± 9.8	0.462^a^
Role-physical	43.1 ± 8.6	44.8 ± 8.9	0.001^a^
Bodily pain	43.9 ± 8.0	44.9 ± 8.5	0.060^a^
General health	49.3 ± 9.3	51.0 ± 8.7	< 0.001^a^
Vitality	54.0 ± 8.5	55.1 ± 8.9	0.133^a^
Social functioning	45.0 ± 9.6	46.4 ± 9.7	0.022^a^
Role-emotional	42.4 ± 9.1	44.6 ± 9.0	< 0.001^a^
Mental health	50.9 ± 7.4	52.3 ± 6.6	0.001^a^
IIQ-7M (IQR)	4.8 (23.8)		< 0.001^b^
Slight UI (0–5)	0 (9.5)		
Moderate UI (6–12)	19.0 (28.6)		
Severe UI (13–21)	16.7 (57.1)		
UDI-6	23.0 ± 14.3		< 0.001^b^
Slight UI (0–5)	18.8 ± 12.4		
Moderate UI (6–12)	27.2 ± 13.9		
Severe UI (13–21)	42.8 ± 17.8		

*SF-12v2* The 12-item Short Form Health Survey version 2, *IIQ-7* Incontinence Impact Questionnaire short form, *M* median, *IQR* inter quartile range, *UI* urinary incontinence, *UDI-6* Urogenital Distress Inventory short form

^a^Adjusted for the following variables: age at first birth, education, pre-pregnancy body mass index, comorbidity (gestational diabetes mellitus)

^b^Adjusted for the following variables: age at first birth, education, pre-pregnancy body mass index, comorbidity (gestational diabetes mellitus), UI type

In the study, only 14.8% of the participants reported help-seeking behavior for urinary leakage. For those who did not seek medical help, the most common reason was the perception that urinary leakage was an inevitable part of pregnancy so they had to put up with it. Besides, many women did not seek professional help because they were not greatly bothered by incontinence or believed that the symptoms could recover by themselves. The help-seeking behavior and detailed reasons for not seeking help were indicated in Table 5.

**Table 5:** Help-seeking behavior of urinary incontinence in pregnant women

Variables	n (%)^a^
Help-seeking behavior	
No	548 (85.2%)
Yes	95 (14.8%)
Reasons for not seeking help for UI	
Regarding UI as an inevitable part of pregnancy	259 (47.3%)
Not greatly bothered by UI	234 (42.7%)
Believing that UI symptoms could recover naturally	166 (30.3%)
Lack of time and energy	32 (5.8%)
Lack of the knowledge of available treatment	22 (4.0%)
Feeling embarrassed to talk about UI	16 (2.9%)
Perception that UI symptoms are not treatable	6 (1.1%)
Having the knowledge of the condition	6 (1.1%)
Postpone help-seeking behavior until after birth	4 (0.7%)
Others	4 (0.7%)

*UI* urinary incontinence

^a^Data of three participants regarding help-seeking behavior were missing

## Discussion

The study comprehensively explored urinary incontinence during pregnancy with universally accepted standardized questionnaires in a relatively large sample, including prevalence, risk factors, its effect on generic and specific quality of life and help-seeking behavior, which could help health care providers get a comprehensive understanding of the occurrence and impact of urinary leakage as well as associated reasons for not seeking medical help from the perspective of the participants.

The prevalence of incontinence was 52.0% in the study, similar to the result in Wesnes’s study but higher than that of a study by Zhu et al. [3, 28]. One explanation was that the study carried out by Zhu et al. only enrolled primiparous women without urinary leakage before pregnancy, while parity and urinary incontinence before pregnancy were confirmed to be predictors for incontinence during pregnancy [3, 6]. Most women in the study suffered from mild or moderate incontinence and stress incontinence was the most common type, which was in line with a recent systematic review [29]. The International Incontinence Society recommended that the degree of bother should be considered in the initial assessment of urinary incontinence. However, bother related to urinary leakage was less assessed especially in pregnant women. We found that the majority of pregnant women were not greatly bothered by incontinence, which was in accordance with a previous study [19]. However, considering the high prevalence and the long-lasting effect of urinary leakage during pregnancy on incontinence in later life, effective preventive strategies should be taken during pregnancy in order to prevent urinary incontinence in postnatal period and later life.

There is evidence that urinary incontinence history plays an important role in the development of incontinence during pregnancy [5, 8, 15]. We found that urinary incontinence before pregnancy was the strongest predictor for incontinence during pregnancy. Compared with nulliparous women, women with a history of vaginal birth were more likely to suffer from incontinence during pregnancy, while a history of cesarean section showed neither protective nor aggravating effect. Similarly, previous studies found that women with previous vaginal birth or instrumental vaginal birth were more susceptible to incontinence during pregnancy [6, 30]. Coffee consumption was associated with an increased risk for incontinence. Results from previous studies were conflicting mainly due to methodological limitations [1]. However, there is a notion that caffeine might incur bladder contraction ahead of time, contributing to the occurrence of urinary incontinence [31]. Our finding supported this hypothesis. Childhood enuresis was found to be associated with urinary incontinence during pregnancy. Although the association between enuresis and incontinence in nulliparous and middle-aged women has been reported, it was first reported in pregnant women. The connection between childhood enuresis and adult incontinence may be attributed to the inability to achieve normal control of urination during toilet training [32]. Pregnant women with a history of urinary tract infection were more likely to report urinary leakage, which was congruent with previous studies [1]. Our findings indicated that lifestyle and behavior modification such as caffeine control and appropriate toilet training might be beneficial for women to prevent incontinence during pregnancy and therefore benefit later life.

Health-related quality of life was less studied in pregnant women and studies incorporating generic quality of life were scarce. In the study, both generic and specific life quality were assessed with universally accepted standardized questionnaires. Poorer quality of life was observed in social functioning, mental health, role-emotional, role-physical and general health in women with urinary leakage, indicating a broad negative effect of incontinence on the life of pregnant women. Moreover, a significant decrease in mental component summary was found in women with incontinence, demonstrating that urinary leakage affected pregnant women more psychologically, which was in line with a previous study regarding stress urinary incontinence during pregnancy [33]. Similarly, in Franco’s study, SF-36 was used to assess generic quality of life in pregnant women and a significant decrease was observed in four domain scales in incontinent women, however, two component summaries were not reported [17]. The specific quality of life perceived by pregnant women was mildly affected by urinary incontinence with a lower score of IIQ-7 and UDI-6, which was similar to previous studies of pregnant women but better than that in general community-dwelling women [4, 33–35]. This finding could be explained by the fact that incontinence symptoms in the majority of pregnant women were mild to moderate and were milder than in general community-dwelling women, while the severity of incontinence symptoms had a significant negative impact on quality of life perceived by participants [15, 16, 34, 35]. Our finding also indicated that the specific quality of life perceived by pregnant women decreased significantly as the incontinence symptoms got worse. The study suggested that more efforts should be made to prevent urinary incontinence during pregnancy and improve the quality of life, especially the mental health, in pregnant women.

The study showed that only 14.8% of pregnant women with incontinence sought professional help, which was similar to a previous study conducted in pregnant women but lower than studies in general adult women [18, 19, 36]. One of the most common reasons for not seeking help from the perspective of the participants in the study was that they were not greatly bothered by urinary leakage, while bother was confirmed to be a strong predictor for help-seeking behavior [37, 38]. Another common reason for not seeking help arose from the misconception that incontinence during pregnancy was inevitable or could recover by itself. Obviously, being a medical disorder, the nature of incontinence and the long-term effect of incontinence during pregnancy on incontinence later in life were not well recognized by pregnant women. It is worth noting that few pregnant women planned to postpone consultation until after childbirth or felt embarrassed to talk about urinary leakage, which provides opportunities for health care providers to talk with pregnant women about urinary leakage initiatively during routine prenatal examinations and inform them about incontinence related knowledge and preventive strategies.

Although the study was conducted in a relatively large sample using universally accepted standardized instruments, several limitations should be noted. First, the participants were recruited from a tertiary hospital, which may limit the generalizability of the results. Second, this was a cross-sectional study, in which recall bias was inevitable and causal inference was not feasible.

## Conclusions

Urinary incontinence was highly prevalent in pregnant women. Urinary incontinence before pregnancy, history of vaginal birth, coffee consumption, childhood enuresis and history of urinary tract infection were confirmed to be associated with increasing the risk of developing urinary incontinence during pregnancy. Although urinary leakage during pregnancy tended to be mild to moderate, it had a broad detrimental effect on health-related quality of life. However, only a few women sought professional help for urinary leakage during pregnancy. Our findings could help health care providers get a comprehensive understanding of the occurrence and impact of urinary leakage as well as the help-seeking behavior in pregnant women, which may facilitate the prevention of urinary incontinence and improvement of health-related quality of life in pregnant women.

## Data Availability

The datasets analyzed during the current study are not publicly available because the data are guaranteed to be confidential.

## Abbreviations

ICIQ-UI SF: International Consultation on Incontinence Modular Questionnaire-Urinary Incontinence Short Form
SF-12v2: 12-Item Short Form Health Survey version 2
UDI-6: Urogenital Distress Inventory short form
IIQ-7: Incontinence Impact Questionnaire short form
UI: Urinary incontinence
M: Median
IQR: Inter quartile range
SF-36: 36-Item Short Form Heath Survey
